# Cancer risks in a population-based study of 70,570 agricultural workers: results from the Canadian census health and Environment cohort (CanCHEC)

**DOI:** 10.1186/s12885-017-3346-x

**Published:** 2017-05-19

**Authors:** Linda Kachuri, M. Anne Harris, Jill S. MacLeod, Michael Tjepkema, Paul A. Peters, Paul A. Demers

**Affiliations:** 1 Dalla Lana School of Public Health, University of Toronto, 155 College Street, 6th Floor, Toronto, ON M5T 3M7 Canada; 2 Occupational Cancer Research Centre, Cancer Care Ontario, 525 University Avenue, 3rd Floor, Toronto, ON M5G 2L3 Canada; 3 Prevention and Cancer Control, Cancer Care Ontario, 620 University Ave, Toronto, ON M5G 2L7 Canada; 4 School of Occupational and Public Health, Ryerson University, 350 Victoria Street, POD 249, Toronto, ON M5B 2K3 Canada; 5 Health Analysis Division, Statistics Canada, 150 Tunney’s Pasture Driveway, Ottawa, ON K1A 0T6 Canada; 6 Departments of Sociology and Economics, University of New Brunswick, Tilley Hall 015, 9 Macaulay Lane, Fredericton, NB E3B 5A3 Canada

## Abstract

**Background:**

Agricultural workers may be exposed to potential carcinogens including pesticides, sensitizing agents and solar radiation. Previous studies indicate increased risks of hematopoietic cancers and decreased risks at other sites, possibly due to differences in lifestyle or risk behaviours. We present findings from CanCHEC (Canadian Census Health and Environment Cohort), the largest national population-based cohort of agricultural workers.

**Methods:**

Statistics Canada created the cohort using deterministic and probabilistic linkage of the 1991 Canadian Long Form Census to National Cancer Registry records for 1992–2010. Self-reported occupations were coded using the Standard Occupational Classification (1991) system. Analyses were restricted to employed persons aged 25–74 years at baseline (*N* = 2,051,315), with follow-up until December 31, 2010. Hazard ratios (HR) and 95% confidence intervals (CI) were modeled using Cox proportional hazards for all workers in agricultural occupations (*n* = 70,570; 70.8% male), stratified by sex, and adjusted for age at cohort entry, province of residence, and highest level of education.

**Results:**

A total of 9515 incident cancer cases (7295 in males) occurred in agricultural workers. Among men, increased risks were observed for non-Hodgkin lymphoma (HR = 1.10, 95% CI = 1.00–1.21), prostate (HR = 1.11, 95% CI = 1.06–1.16), melanoma (HR = 1.15, 95% CI = 1.02–1.31), and lip cancer (HR = 2.14, 95% CI = 1.70–2.70). Decreased risks in males were observed for lung, larynx, and liver cancers. Among female agricultural workers there was an increased risk of pancreatic cancer (HR = 1.36, 95% CI = 1.07–1.72). Increased risks of melanoma (HR = 1.79, 95% CI = 1.17–2.73), leukemia (HR = 2.01, 95% CI = 1.24–3.25) and multiple myeloma (HR = 2.25, 95% CI = 1.16–4.37) were observed in a subset of female crop farmers.

**Conclusions:**

Exposure to pesticides may have contributed to increased risks of hematopoietic cancers, while increased risks of lip cancer and melanoma may be attributed to sun exposure. The array of decreased risks suggests reduced smoking and alcohol consumption in this occupational group compared to the general population.

**Electronic supplementary material:**

The online version of this article (doi:10.1186/s12885-017-3346-x) contains supplementary material, which is available to authorized users.

## Background

Agricultural workers represent a unique population. While these individuals are employed in a range of occupations associated with exposure to a number of potential carcinogens, they also have a lower prevalence of cigarette smoking and increased levels of physical activity [1]. As a result, individuals employed in agriculture tend to experience lower overall morbidity and mortality compared to the general population, and also exhibit distinctive cancer risk profiles [1–4]. Studies of cancer incidence and mortality in farmers have consistently reported reduced risks of tobacco-related cancers, such as lung and bladder [2, 3, 5–7]. Lower incidence of colorectal cancer has also been attributed to this favourable risk factor profile that includes higher levels of physical activity [3, 5, 7–9].

However, studies of agricultural workers also point to a number of elevated cancer risks in this population, especially for lymphatic and hematopoietic cancers, melanoma, lip cancer, prostate cancer and brain tumors [2, 3, 5, 7]. This pattern is consistent with hazards associated with occupational exposure to pesticides, solvents, sunlight, dusts, and biological agents, such as zoonotic viruses, fungi and bacteria. Recent reports from the Agricultural Health Study (AHS), a large cohort of pesticide applicators and their spouses, also confirm many previous findings and implicate certain pesticides as key determinants of the cancer risks observed in agricultural populations [10].

Despite the accumulating evidence for a unique pattern of cancer incidence among agricultural workers and farmers, the last meta-analysis of studies in this area, conducted in 1998, reported inconsistent and highly heterogeneous results [3]. In addition to the multiplicity of exposures in agriculture, a possible explanation for these findings is that many earlier studies of agricultural populations have used mortality as the primary outcome, making it difficult to disentangle factors that influence cancer development from prognosis. Since then, few large-scale population-based occupational studies have been conducted, with one notable exception being the Nordic Occupational Cancer Study (NOCCA), which reported cancer incidence patterns among farmers and other occupational subgroups have been carried out in Denmark, Finland, Iceland, Norway and Sweden [5, 7]. However, similar comprehensive studies were lacking in Canada, despite the fact that agriculture has historically been one of the largest sectors of the Canadian economy.

A narrative review of the literature on cancer among farmers and an editorial dedicated to the NOCCA project, both published in 2009, called for future studies focusing on specific exposures, as well as the need for continued occupational cancer surveillance to document cancer incidence in agricultural workers and other occupational subgroups. [11, 12]. Furthermore, a consortium of agricultural cohort studies, AGRICOH, has been formed in 2010 to promote research efforts in this area, such as pooling of data across prospective studies to enable large-scale analyses of specific exposure-disease associations [13].

The Canadian Census Health and Environment Cohort (CanCHEC) was assembled to investigate cancer incidence patterns in specific socio-demographic and occupational groups in Canada. The aim of the present study was to address an important gap in Canadian occupational cancer surveillance by providing a comprehensive analysis of cancer risks among men and women employed in agriculture, by using data from a representative sample of the Canadian population. To our knowledge, this is the first and largest study of its kind in Canada, enabling a systematic analysis of cancer incidence by occupational subgroup, on a scale not previously possible in a single study.

## Methods

### Study population

The 1991–2010 Canadian Census Mortality and Cancer Follow-Up cohort is the foundational parent study for the Canadian Census Health and Environment Cohort (CanCHEC) [14, 15]. This cohort was originally created by linking the 1991 Canadian Census 2B (long form), with the Canadian Mortality Database (CMDB) and annual Historic Tax Summary Files (HTSF), to enable mortality follow-up from 1991 to 2001 [14, 15]. Individuals were eligible for the cohort if they were 25 years of age or older on Census day (June 4, 1991), were a usual resident of Canada, were not a long-term resident of an institution such as a prison, hospital or nursing home, and were among the 20% of Canadian households selected to complete the long-form census questionnaire.

The same inclusion criteria were maintained for CanCHEC, which was created by expanding the linkage of the 1991 Canadian Census to the Canadian Cancer Database (CCDB), in addition to updating the CMDB (1991–2011) and HTSF (1984–2011) data to 2011. Deterministic and probabilistic matching methods were used to link in-scope 1991 Census records to non-financial HTSF data, using dates of birth and postal codes of the individual and, if applicable, his or her spouse or common-law partner. The purpose of linkage to the HTSF files was to ascertain loss to follow-up, for instance due to a move outside of Canada. Individuals who stopped filing income taxes for 4 or more consecutive years by the end of the follow-up period, on December 31 2010, were identified using the HTSF and were considered lost to follow-up.

A total of 2.7 million individuals were successfully linked to the CMDB, equal to 15% of the Canadian non-institutional resident population aged 25 years or older on Census day in 1991 [15]. Follow-up started on Census day in 1991 (June 4, 1991) and continued until December 31 2010. Demographic and socioeconomic information, and including age, sex, province or territory of residence, highest level of education, and occupation and industry were obtained from the 1991 long-form census.

### Ascertainment of cancer diagnosis

Cancer diagnoses in CanCHEC were ascertained using data from the Canadian Cancer Database (CCDB). The CCDB combines two cancer data sources: the Canadian Cancer Registry (CCR) and the National Cancer Incidence Reporting System (NCIRS). The CCR is a person-oriented tumor database that records the incidence of primary cancers diagnosed for each person since 1992 [16]. Subsequent primary cancers diagnosed in patients who are already in the database are linked to their existing information. The NCIRS is a historical tumor-oriented database, which contains information on cancers diagnoses as far back as 1969 [16]. Individual cancer records from the CCR are used in the analysis, whereas historical information from NCIRS, linked to the CCR using probabilistic methods, was used to identify and exclude individuals with a cancer diagnosis within 10 years of cohort inception (1981–1991) in sensitivity analyses.

In our study, a case was defined as a primary incident cancer diagnosed between January 1, 1992 and December 31, 2010. Information on incident cancer diagnosis and year of diagnosis was retrieved from the CCR, and year of death was obtained from the CMDB, in order to remove deceased individuals from the cohort and ensure that the date of cancer diagnosis preceded the date of death. All cancers were defined using the 3rd revision of the International Classification of Diseases for Oncology (ICD-O-3) codes. With the exception of hematologic cancers (non-Hodgkin lymphoma, Hodgkin lymphoma, multiple myeloma, and leukemia) and mesothelioma, which were defined using morphology codes, the remaining cancer sites were identified using topography codes. Multiple cancers in the same individual were counted according to the International Agency for Research on Cancer (IARC) multiple primary rules [17].

This analysis examined a total of 25 diagnostic categories among male and female agricultural workers. For an assessment of overall cancer incidence, the “any cancer” category included all neoplasms, excluding non-melanoma skin cancer. For individuals with multiple primary cancers, only the first diagnosis was counted in the any cancer category.

### Exposure assessment

In the 1991 Census a respondent’s occupation was determined by the job held in the week prior to the Census. If a participant reported no job in the last week, then the job with the longest duration since January 1, 1990 was recorded. If a participant had more than one job, then the job where most hours were worked was recorded as the main occupation.

Occupational data collected by Statistics Canada follows the structural framework of the Standard Occupational Classification (SOC). All occupations reported by the census participants were coded at Statistics Canada using the SOC 1991 system, and this forms the basis of the exposure assessment in our analysis. Within SOC91, occupations are grouped into 10 general categories, denoted by letters A – J, and occupational groups are further refined using 1–3 numbers. A total of 512 occupation groups are defined by the SOC 1991, which is based closely on its predecessor, the SOC 1980.

The specific SOC91 codes used to assess agricultural occupations and define exposure are presented in Table 1. In order to examine potential differences in risk between individuals employed in occupations that involve manual labour, the main exposure group was separated into two categories: farmers and managers (SOC91: I011-I016), and manual labourers (SOC91: I021, I022, I211, I212). In an effort to further refine the exposure groups by distinguishing between different types of agricultural work, classifications based on the SOC80 coding system were used to identify crop farmers and workers (SOC80: 7115, 7185) and livestock farmers and workers (SOC80: 7113, 7183).

**Table 1:** Description of occupations used to define agricultural workers in CanCHEC (1991–2010)

Standard Occupational Classification (SOC) 1991 and 1980 Codes	Males		Females		Total	
	N	(%)	N	(%)	N	(%)
Farmers and managers (SOC91)	33,980	(68.0)	9385	(45.5)	43,365	(61.4)
Farmers and farm managers (I011)	30,215	(60.5)	8415	(40.8)	38,630	(54.7)
Agricultural and related contractors and managers (I012)	215	(0.4)	20	(0.1)	235	(0.3)
Farm supervisors and specialized livestock workers (I013)	960	(1.9)	315	(1.5)	1275	(1.8)
Nursery and greenhouse operators and managers (I014)	495	(1.0)	355	(1.7)	850	(1.2)
Landscaping, ground maintenance contractors and managers (I015)	925	(1.9)	115	(0.6)	1040	(1.5)
Landscape and horticulture supervisors (I016)	1170	(2.3)	165	(0.8)	1335	(1.9)
Manual labourers (SOC91)	15,985	(32.0)	11,220	(54.5)	27,205	(38.6)
General farm workers (I021)	8945	(17.9)	7620	(37.0)	16,565	(23.5)
Nursery and greenhouse workers (I022)	1465	(2.9)	1785	(8.7)	3250	(4.6)
Harvesting labourers (I211)	485	(1.0)	1020	(5.0)	1505	(2.1)
Landscaping and grounds maintenance labourers (I212)	5090	(10.2)	795	(3.9)	5885	(8.4)
Specialized subgroups (SOC80)						
Crop farmers and farm workers (7115, 7185)	2235	(4.5)	3505	(17.0)	5740	(8.1)
Livestock farmers and farm workers (7113, 7183)	2300	(4.6)	1740	(8.4)	4040	(5.7)
Total	49,965	(100.0)	20,605	(100.0)	70,570	(100.0)

Note: counts below 5 have been suppressed and all counts have been randomly rounded to base 5 in accordance with Statistics Canada disclosure rules

To minimize the healthy worker effect in CanCHEC, a working cohort was created by excluding individuals without a valid entry for occupation on the 1991 long-form census [18]. This working cohort was further refined to minimize survival bias by excluding individuals over the age of 74 at baseline on June 4, 1991.

### Statistical analysis

Hazard ratios (HRs) and corresponding 95% confidence intervals (CI) were calculated using Cox proportional hazards regression to estimate cancer risks associated with employment in agriculture. Cox regression was selected over more crude approaches, such as standardization, because it allows for covariate adjustment, and unlike Poisson regression, the Cox model does not make parametric assumptions about the baseline hazard [19, 20].

The reference group consisted of all other employed cohort members, specifically, individuals not captured by the occupational groups in Table 1. Models were adjusted for age at cohort entry (age group categories: 25–34, 35–44, 45–54, 55–74) and province of residence at the time of the Census. To control for potential confounding by socio-economic status, differences in screening rates, and lifestyle factors including cigarette smoking, physical activity, and diet, models were adjusted for highest attained level of education, which has been proposed as a suitable measure of socio-economic status and proxy for life-style related risk factors [21, 22]. We verified that none of the predictors violated the proportionality of hazards assumption. Stratified analyses were carried out to examine risks separately among men and women.

For each member of the cohort, follow-up time accrued from cohort entry on June 4, 1991 to date of cancer diagnosis, date of death, date of loss to follow-up or end of follow-up on December 31, 2010, whichever occurred first. Deceased individuals were identified in the CMDB and removed from the at risk population in the cohort. Person-days were divided by 365.25 to obtain person-years at risk.

Data were accessed and analyzed in the secure facilities of the Toronto Research Data Centre located at Robarts Library, University of Toronto. Statistical analyses were performed using SAS 9.3/9.4 statistical software (SAS Institute Inc., Cary, NC, USA).

In accordance with Statistics Canada disclosure rules, case counts of less than 5 were suppressed in the reported tables, all frequencies were randomly rounded to the base 5, and reported person-years at risk were rounded to the nearest 10.

## Results

### Population characteristics

**Fig. 1:**
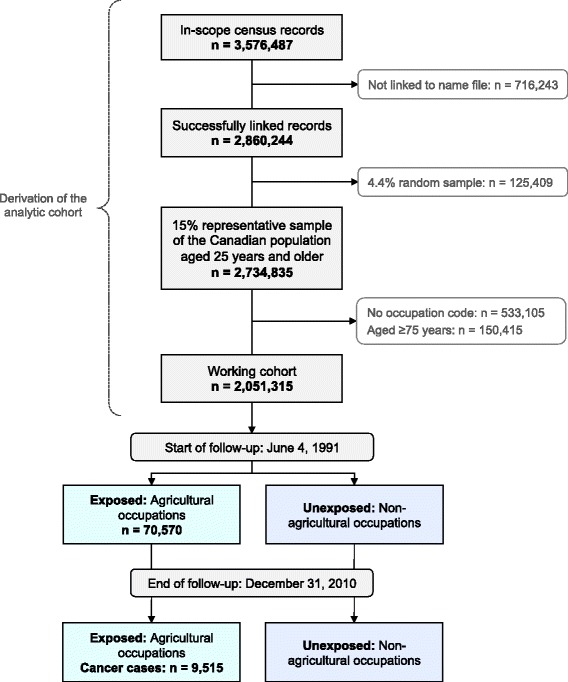
Canadian Census Health and Environment Cohort (CanCHEC) participant flow and sample size based on the analytic cohort of derived from 1991 Census records

The derivation of the analytic cohort and flow of participants during the study period are illustrated in Fig. 1. Using the SOC91 occupation codes, we identified a total of 70,570 individuals (49,965 men, 20,605 women) employed in agriculture (Table 1). Farmers and managers constituted 61.4% of the main exposure group with 43,365 individuals, while the remaining 27,205 subjects (38.6%) were classified as manual laborers. Examining this distribution by gender revealed that most male agricultural workers (68.0%, 33,980 subjects) were farmers and managers, whereas the majority of women (54.5%, 11,220 subjects) worked in occupations associated with manual labour.

Using the SOC80 system, 5740 crop farmers and workers (17% of female agricultural workers, 4.5% of male agricultural workers) and 4040 livestock farmers and workers (5.7% of females, 4.6% of males) were also identified. Crop farmers and workers accounted for 47% of general farm workers (SOC91: I021), 20% of farmers and farm managers (SOC91: I011), 16% of landscaping and grounds maintenance labourers (SOC91: I212), and 10% of nursery and greenhouse workers (SOC91: I022). The majority of livestock farmers and workers (58%) were included in the SOC91 general farm workers group (I021), followed by 28% in the farmers and farm managers category (SOC91: I011), and 6% were part of the farm supervisors and specialized livestock workers (SOC91: I013) group. Overall, 92% of crop farmers and workers and 93% of livestock farmers and workers identified using SOC80 were also captured by the SOC91 agricultural occupations.

Baseline demographic characteristics of CanCHEC are presented in Table 2. Compared to the entire working cohort, agricultural workers were older, predominantly male (70.8%), and had a lower proportion of individuals with a university degree. Between cohort inception in 1991 and end of follow-up in December 2010, agricultural workers had accrued a total of 789,390 person-years of follow-up in males and 448,205 person-years in females. The mean person-years of follow-up were similar between agricultural workers (17.1 males, 17.9 females) and the entire working CanCHEC sample (17.7 for males, 18.2 females).

**Table 2:** Baseline characteristics of CanCHEC (1991–2010) subjects aged 25 to 74 years at baseline, by employment status

Characteristics	Agricultural Workers (*n* = 70,570)				Working Cohort (*n* = 2,051,315)			
	Males		Females		Males		Females	
	N	(%)	N	(%)	N	(%)	N	(%)
Age at entry to cohort								
Mean age in years (SD)	46.4	(13.7)	44.8	(12.5)	41.7	(11.3)	40.2	(10.5)
25–34 years	12,840	(25.7)	5355	(26.0)	359,075	(32.4)	336,870	(35.7)
35–44 years	11,640	(23.3)	5500	(26.7)	341,515	(30.8)	309,600	(32.8)
45–54 years	9245	(18.5)	4640	(22.5)	229,460	(20.7)	187,465	(19.9)
55–65 years	10,195	(20.4)	3520	(17.1)	143,895	(13.0)	91,135	(9.7)
65–74 years	6045	(12.1)	1590	(7.7)	34,465	(3.1)	17,845	(1.9)
Province of residence								
Newfoundland and Labrador	250	(0.5)	80	(0.4)	21,815	(2.0)	17,530	(1.9)
Prince Edward Island	500	(1.0)	165	(0.8)	4945	(0.4)	4475	(0.5)
Nova Scotia	900	(1.8)	370	(1.8)	34,750	(3.1)	28,405	(3.0)
New Brunswick	800	(1.6)	350	(1.7)	27,600	(2.5)	22,615	(2.4)
Quebec	7845	(15.7)	3070	(14.9)	276,120	(24.9)	226,375	(24.0)
Ontario	12,290	(24.6)	5375	(26.1)	404,130	(36.5)	352,165	(37.3)
Manitoba	4545	(9.1)	1525	(7.4)	47,375	(4.3)	40,190	(4.3)
Saskatchewan	9395	(18.8)	3320	(16.1)	42,050	(3.8)	35,805	(3.8)
Alberta	9295	(18.6)	3875	(18.8)	107,405	(9.7)	92,730	(9.8)
British Columbia	4045	(8.1)	2455	(11.9)	130,815	(11.8)	112,920	(12.0)
Yukon, Northwest Territories, Nunavut	100	(0.2)	20	(0.1)	11,395	(1.0)	9705	(1.0)
Highest level of education								
No high school diploma	27,080	(54.2)	9785	(47.5)	322,190	(29.1)	233,565	(24.8)
High school or trade certificate	16,440	(32.9)	7150	(34.7)	444,560	(40.1)	360,215	(38.2)
Post-secondary non-university	4345	(8.7)	2845	(13.8)	154,165	(13.9)	207,865	(22.0)
University degree	2100	(4.2)	825	(4.0)	187,495	(16.9)	141,260	(15.0)
Subtotal	49,965	(70.8)^a^	20,605	(29.2)^a^	1,108,410	(54.0)^b^	942,905	(46.0)^b^
Person-years of follow-up Total (Mean)	789,390	(17.1)	448,205	(17.9)	19,635,045	(17.7)	17,116,840	(18.2)

Note: case counts below 5 have been suppressed and all counts have been randomly rounded to base 5 in accordance with Statistics Canada disclosure rules

^a^Percentage calculated from total number of agricultural workers (*n* = 70,570)

^b^Percentage calculated from total number of subjects in working cohort (*n* = 2,051,315)

Over the course of the follow-up period, a total of 9515 primary incident cancer cases were observed among agricultural workers. Of these, 7295 cases (76.7%) were observed in men, who account for 70.8% of all agricultural workers, and 2220 cancers (23.3%) occurred in women, who represent 29.2% of individuals employed in agriculture.

### Cancer risks in men

Compared to the reference group, comprised of the working CanCHEC population, male agricultural workers were less likely to develop any type of malignancy (HR = 0.95, 95% CI = 0.93–0.98) (Table 3). This inverse relationship remained statistically significant among farmers and managers (HR = 0.94, 95% CI = 0.92–0.97), the larger occupational subgroup for male agricultural workers, and crop farmers (HR = 0.84, 95% CI = 0.75–0.95).

**Table 3:** Hazard ratios (HR) and 95% confidence intervals (CI) for selected cancers among male agricultural workers in CanCHEC (1991–2010)

Cancer Site (ICD-O-3)	Agricultural Workers			Farmers and managers			Manual labourers		
	Cases	HR	(95% CI)^a^	Cases	HR	(95% CI)^a^	Cases	HR	(95% CI)^a^
Any cancer^b^	7295	0.95	(0.93–0.98)	5415	0.94	(0.92–0.97)	1880	0.98	(0.93–1.02)
Prostate (C61.9)	2625	1.11	(1.06–1.16)	2025	1.12	(1.07–1.17)	600	1.08	(0.99–1.17)
Lung (C34)	995	0.75	(0.70–0.80)	690	0.69	(0.64–0.75)	305	0.90	(0.81–1.01)
Colon (C18, C26.0)	640	0.89	(0.82–0.97)	485	0.90	(0.82–0.99)	155	0.88	(0.75–1.03)
Rectum (C19.9, C20.9)	440	1.05	(0.95–1.16)	340	1.09	(0.97–1.22)	100	0.94	(0.77–1.15)
Non-Hodgkin Lymphoma^c^	500	1.10	(1.00–1.21)	375	1.11	(0.99–1.24)	125	1.09	(0.91–1.30)
Bladder (C67)	430	0.82	(0.74–0.91)	325	0.83	(0.73–0.93)	105	0.82	(0.67–1.00)
Melanoma (C44)	275	1.15	(1.02–1.31)	215	1.21	(1.05–1.39)	60	1.00	(0.77–1.29)
Leukemia^c^	265	1.11	(0.97–1.27)	200	1.14	(0.98–1.32)	65	1.03	(0.80–1.32)
Oral (C00-C14)	250	1.04	(0.91–1.19)	170	0.97	(0.82–1.14)	80	1.24	(0.99–1.55)
Lip (C00.0-C00.9)	110	2.14	(1.70–2.70)	90	2.25	(1.75–2.90)	20	1.80	(1.15–2.84)
Kidney (C64.9)	230	0.77	(0.67–0.89)	175	0.78	(0.67–0.92)	55	0.74	(0.57–0.97)
Stomach (C16)	225	0.90	(0.78–1.04)	160	0.87	(0.74–1.03)	65	0.99	(0.77–1.26)
Pancreas (C25)	175	0.90	(0.77–1.06)	125	0.86	(0.71–1.03)	50	1.03	(0.77–1.36)
Multiple myeloma^c^	135	1.15	(0.95–1.38)	100	1.14	(0.92–1.41)	35	1.17	(0.84–1.64)
Brain (C70-C72)	110	0.89	(0.73–1.08)	75	0.84	(0.66–1.06)	35	1.01	(0.72–1.41)
Esophagus (C15)	95	0.87	(0.70–1.08)	65	0.78	(0.60–1.00)	30	1.15	(0.80–1.65)
Thyroid (C73.9)	60	1.05	(0.79–1.38)	45	1.06	(0.77–1.47)	15	1.00	(0.59–1.70)
Larynx (C32)	55	0.54	0.41–0.71)	30	0.39	(0.27–0.56)	25	0.96	(0.64–1.43)
Liver (C22.0, C22.1)	45	0.51	(0.38–0.68)	30	0.43	(0.30–0.62)	15	0.75	(0.47–1.20)
Testis (C62)	30	0.97	(0.69–1.37)	20	1.02	(0.66–1.56)	10	0.90	(0.52–1.56)
Hodgkin Lymphoma^c^	25	0.78	(0.49–1.25)	15	0.66	(0.36–1.22)	10	1.02	(0.51–2.07)
Mesothelioma^c^	20	0.57	(0.36–0.90)	15	0.56	(0.33–0.95)	5	0.59	(0.24–1.43)
Breast (C50)	20	1.27	(0.74–2.15)	10	0.96	(0.48–1.91)	10	2.08	(0.97–4.44)
Nasal (C30)	15	0.71	(0.39–1.29)	10	0.77	(0.38–1.53)	5	0.60	(0.22–1.65)
Bone (C40, C41)	5	0.69	(0.36–1.32)	<5	-	-	<5	-	-

Note: case counts below 5 have been suppressed and all counts have been randomly rounded to base 5 in accordance with Statistics Canada disclosure rules

^a^Adjusted for age at baseline (age group categories), province of residence at baseline, and education level at baseline

^b^Incident primary cancers excluding non-melanoma skin cancer

^c^Cancers defined using ICD-O-3 Histology codes: Mesothelioma (9050–9055), Hodgkin lymphoma (9650–9667); non-Hodgkin lymphoma (9590–9596,9670–9719, 9727–9729, 9823, 9827); Multiple myeloma (9731,9732,9734); Leukemia (9733, 9742, 9800–9801, 9805, 9820, 9826, 9831–9837, 9840, 9860–9861, 9863, 9866–9867, 9870–9876,9891,9895–9897,9910, 9920, 9930–9931, 9940, 9945–9946, 9948, 9963–9964, 9823, 9827)

An array of decreased risks associated with employment in agriculture was observed across several sites, especially for cancers strongly linked to cigarette smoking and alcohol consumption (Table 3). Statistically significant inverse risk estimates were observed for cancer of the colon, liver, bladder, kidney, larynx, lung, and mesothelioma.

However, a number of statistically significant excesses in cancer risk were also observed among male agricultural workers in our cohort. The risk of being diagnosed with lip cancer was almost doubled for all agricultural workers (HR = 2.14, 95% CI = 1.70–2.70), farmers and managers (HR = 2.25, 95% CI = 1.75–2.90), and manual labourers (HR = 1.80, 95% CI = 1.15–2.84). A statistically significant increase in melanoma risk was also observed among all males in agriculture (HR = 1.15, 95% CI = 1.02–1.31), and farmers and managers (HR = 1.21, 95% 1.05–1.39).

Other notable increases in risk were observed for prostate cancer and non-Hodgkin lymphoma (NHL). The increase in the risk of prostate cancer was observed for all agricultural workers (HR = 1.11, 95% CI = 1.06–1.16), farmers and managers (HR = 1.12, 95% CI = 1.07–1.17), and manual labourers (HR = 1.08, 95% CI = 0.99–1.17). The increase in NHL risk was similar in magnitude, but reached marginal significance only in the overall exposure group (HR = 1.10, 95% CI = 1.00–1.21).

Investigating more refined agricultural subgroups revealed several notable increases in risk among crop farmers (Table 5) and livestock farmers (Table 6). Mirroring the main analyses, risks of lip cancer remained elevated for both crop (HR = 3.59, 95% CI = 1.69–7.62) and livestock farmers (HR = 2.91, 95% CI = 1.08–7.80). The risk of prostate cancer was elevated only in the livestock subgroup (HR = 1.26, 95% CI = 1.03–1.55). Despite a small number of cases, a large and statistically significant increase in thyroid cancer risk (HR = 3.01, 95% CI = 1.35–6.73) was also observed among livestock farmers.

### Cancer risks in women

Similar to the pattern observed for men, women employed in agriculture appeared to have a decreased overall cancer risk (HR = 0.92 95% CI = 0.88–0.96). The inverse association between agricultural occupation and cancer risk remained statistically significant in the larger manual laborers subgroup (HR = 0.88, 95% CI = 0.83–0.93), and among crop farmers (HR = 0.85, 95% CI = 0.76–0.94).

In contrast to the multiple negative associations observed in men, only lung cancer exhibited inverse risk estimates among women across most exposure groups (Table 4). This negative association was statistically significant for all agricultural occupations (HR = 0.58, 95% CI = 0.50–0.66), farmers and managers (HR = 0.57, 95% CI = 0.47–0.70), and manual labourers (HR = 0.58, 95% CI = 0.48–0.70). In addition, belonging to the manual labourer subgroup was also associated with a decreased risk of colon cancer (HR = 0.73, 95% CI = 0.58–0.91). Notably, the pattern of colon cancer risk among farmers and managers was in the opposite direction (HR = 1.19, 95% CI = 0.99–1.42) in women compared to men.

**Table 4:** Hazard ratios (HR) and 95% confidence intervals (CI) for selected cancers among female agricultural workers in CanCHEC (1991–2010)

Cancer Site (ICD-O-3)	Agricultural Workers			Farmers and managers			Manual labourers		
	Cases	HR	(95% CI)^a^	Cases	HR	(95% CI)^a^	Cases	HR	(95% CI)^a^
Any cancer^b^	2220	0.92	(0.88–0.96)	1130	0.96	(0.90–1.02)	1090	0.88	(0.83–0.93)
Breast (C50)	700	0.92	(0.85–0.99)	350	0.92	(0.83–1.03)	350	0.92	(0.83–1.02)
Lung (C34)	210	0.58	(0.50–0.66)	100	0.57	(0.47–0.70)	110	0.58	(0.48–0.70)
Colon (C18, C26.0)	205	0.95	(0.82–1.10)	120	1.19	(0.99–1.42)	80	0.73	(0.58–0.91)
Rectum (C19.9, C20.9)	100	1.08	(0.88–1.32)	45	1.08	(0.81–1.45)	55	1.07	(0.81–1.42)
Non-Hodgkin Lymphoma^c^	135	1.02	(0.86–1.22)	75	1.17	(0.92–1.47)	65	0.89	(0.69–1.15)
Melanoma (C44)	90	1.14	(0.93–1.41)	40	1.00	(0.72–1.39)	55	1.26	(0.96–1.66)
Ovary (C56.9)	80	0.99	(0.79–1.23)	75	0.90	(0.65–1.25)	45	1.07	(0.79–1.43)
Leukemia^c^	75	1.21	(0.95–1.54)	45	1.70	(1.26–2.29)	30	0.82	(0.56–1.20)
Thyroid (C73.9)	75	1.25	(0.99–1.59)	35	1.25	(0.88–1.77)	40	1.26	(0.92–1.72)
Pancreas (C25)	75	1.36	(1.07–1.72)	40	1.27	(0.91–1.77)	35	1.45	(1.05–2.01)
Bladder (C67)	50	0.88	(0.65–1.17)	20	0.86	(0.56–1.32)	30	0.89	(0.60–1.31)
Kidney (C64.9)	45	0.79	(0.58–1.08)	25	0.88	(0.59–1.32)	15	0.70	(0.44–1.12)
Brain (C70-C72)	45	1.26	(0.92–1.71)	20	1.42	(0.94–2.16)	20	1.11	(0.71–1.73)
Cervix (C53)	40	0.83	(0.60–1.15)	10	0.69	(0.41–1.18)	25	0.94	(0.63–1.41)
Stomach (C16)	35	0.92	(0.65–1.31)	20	1.14	(0.73–1.79)	10	0.71	(0.41–1.22)
Oral (C00-C14)	35	0.79	(0.55–1.13)	15	0.80	(0.50–1.29)	15	0.78	(0.46–1.32)
Lip (C00.0-C00.9)	<5	-	-	<5	-	-	<5	-	-
Multiple Myeloma^c^	25	0.81	(0.54–1.21)	10	0.54	(0.30–0.99)	15	1.24	(0.74–2.08)
Liver (C22.0, C22.1)	15	0.90	(0.51–1.57)	5	0.88	(0.41–1.88)	5	0.92	(0.41–2.06)
Esophagus (C15)	10	1.06	(0.60–1.87)	10	1.40	(0.65–2.98)	5	0.82	(0.36–1.88)
Mesothelioma^c^	5	1.14	(0.37–3.47)	<5	-	-	<5	-	-
Nasal (C30)	<5	-	-	<5	-	-	<5	-	-
Larynx (C32)	<5	-	-	<5	-	-	<5	-	-
Bone (C40, C41)	<5	-	-	<5	-	-	<5	-	-
Hodgkin Lymphoma^c^	<5	-	-	<5	-	-	<5	-	-

Note: case counts below 5 have been suppressed and all counts have been randomly rounded to base 5 in accordance with Statistics Canada disclosure rules

^a^Adjusted for age at baseline (age group categories), province of residence at baseline, and education level at baseline

^b^Incident primary cancers excluding non-melanoma skin cancer

^c^Cancers defined using ICD-O-3 Histology codes: Mesothelioma (9050–9055), Hodgkin lymphoma (9650–9667); non-Hodgkin lymphoma (9590–9596,9670–9719, 9727–9729, 9823, 9827); Multiple myeloma (9731,9732,9734); Leukemia (9733, 9742, 9800–9801, 9805, 9820, 9826, 9831–9837, 9840, 9860–9861, 9863, 9866–9867, 9870–9876,9891,9895–9897,9910, 9920, 9930–9931, 9940, 9945–9946, 9948, 9963–9964, 9823, 9827)

Few statistically significant increases in risk were observed among female agricultural workers (Table 4). The incidence of pancreatic cancer was significantly higher among all females employed in agriculture (HR = 1.36, 95% CI = 1.07–1.72), and manual labourers (HR = 1.45, 95% CI = 1.05–2.01). Risk of leukemia was also significantly elevated among farmers and managers (HR = 1.70, 95% CI = 1.26–2.29).

Elevated risks that did not reach statistical significance were observed for thyroid cancer in all female agricultural workers (HR = 1.25, 95% CI = 0.99–1.59), melanoma among manual labourers (HR = 1.26, 95% CI = 0.96–1.66), and brain tumors in the farmer and manager subgroup (HR = 1.42, 95% CI = 0.94–2.16).

Several associations emerged from more refined analyses of crop farmers (Table 5) and livestock farmers (Table 6). Crop farmers had an approximately 2-fold increase in the risk of multiple myeloma (HR = 2.25, 95% CI = 1.16–4.37), leukemia (HR = 2.01, 95% CI = 1.24–3.25), and melanoma (HR = 1.79, 95% CI = 1.17–2.73). Although based on a small number of cases, significant excesses in oral cancer risk were observed among women working as livestock farmers (HR = 2.70, 95% CI = 1.28–5.68).

**Table 5:** Hazard ratios (HR) and 95% confidence intervals (CI) for selected cancers among male and female crop farmers and farm workers in CanCHEC (1991–2010)

Cancer Site (ICD-O-3)	Men			Women		
	Cases	HR	(95% CI)^a^	Cases	HR	(95% CI)^a^
Any cancer^b^	270	0.84	(0.75–0.95)	355	0.85	(0.76–0.94)
Breast (C50)	0	-	-	110	0.85	(0.70–1.02)
Prostate (C61.9)	80	0.82	(0.66–1.02)	-	-	-
Leukemia3	80	1.08	(0.60–1.95)	15	2.01	(1.24–3.25)
Lung (C34)	50	0.85	(0.64–1.13)	30	0.44	(0.31–0.63)
Melanoma (C44)	<5	-	-	25	1.79	(1.17–2.73)
Colon (C18, C26.0)	30	1.10	(0.77–1.56)	20	0.62	(0.39–0.97)
Rectum (C19.9, C20.9)	15	0.88	(0.53–1.46)	15	0.96	(0.58–1.59)
Non-Hodgkin Lymphoma^c^	15	1.16	(0.48–2.80)	15	0.74	(0.47–1.18)
Bladder (C67)	15	0.75	(0.47–1.20)	<5	-	-
Thyroid (C73.9)	0	-	-	15	1.55	(0.96–2.51)
Ovary (C56.9)	-	-	-	15	1.08	(0.66–1.77)
Cervix (C53)	-	-	-	10	1.13	(0.59–2.18)
Multiple Myeloma^c^	10	0.84	(0.52–1.38)	10	2.25	(1.16–4.37)
Kidney (C64.9)	10	0.90	(0.48–1.68)	10	0.82	(0.39–1.74)
Stomach (C16)	10	0.61	(0.32–1.18)	10	1.05	(0.50–2.21)
Oral (C00-C14)	10	1.26	(0.71–2.22)	5	0.82	(0.34–1.98)
Lip (C00.0-C00.9)	10	3.59	(1.69–7.62)	0	-	-
Pancreas (C25)	5	0.87	(0.41–1.83)	5	1.02	(0.53–1.96)
Brain (C70-C72)	<5	-	-	5	1.06	(0.47–2.37)
Larynx (C32)	5	1.50	(0.67–3.36)	0	-	-
Bone (C40, C41)	<5	-	-	<5	-	-
Liver (C22.0, C22.1)	<5	-	-	0	-	-
Nasal (C30)	<5	-	-	0	-	-
Esophagus (C15)	0	-	-	<5	-	-
Mesothelioma^c^	0	-	-	<5	-	-
Hodgkin Lymphoma^c^	0	-	-	<5	-	-
Testis (C62)	0	-	-	-	-	-

Note: case counts below 5 have been suppressed and all counts have been randomly rounded to base 5 in accordance with Statistics Canada disclosure rules

^a^Adjusted for age at baseline (age group categories), province of residence at baseline, and education level at baseline

^b^Incident primary cancers excluding non-melanoma skin cancer

^c^Cancers defined using ICD-O-3 Histology codes: Mesothelioma (9050–9055), Hodgkin lymphoma (9650–9667); non-Hodgkin lymphoma (9590–9596,9670–9719, 9727–9729, 9823, 9827); Multiple myeloma (9731,9732,9734); Leukemia (9733, 9742, 9800–9801, 9805, 9820, 9826, 9831–9837, 9840, 9860–9861, 9863, 9866–9867, 9870–9876,9891,9895–9897,9910, 9920, 9930–9931, 9940, 9945–9946, 9948, 9963–9964, 9823, 9827)

**Table 6:** Hazard ratios (HR) and 95% confidence intervals (CI) for selected cancers among male and female livestock farmers and farm workers in CanCHEC (1991–2010)

Cancer Site (ICD-O-3)	Men			Women		
	Cases	HR	(95% CI)^a^	Cases	HR	(95% CI)^a^
Any cancer^b^	240	0.94	(0.83–1.07)	160	0.90	(0.77–1.05)
Prostate (C61.9)	90	1.26	(1.03–1.55)	-	-	-
Breast (C50)	0	-	-	45	0.76	(0.57–1.02)
Lung (C34)	30	0.69	(0.48–0.98)	20	0.61	(0.37–0.99)
Colon (C18, C26.0)	20	0.97	(0.63–1.49)	20	1.35	(0.87–2.09)
Rectum (C19.9, C20.9)	10	0.93	(0.54–1.60)	5	0.86	(0.36–2.06)
Non-Hodgkin Lymphoma^c^	15	0.89	(0.54–1.45)	5	0.72	(0.33–1.61)
Melanoma (C44)	15	1.44	(0.82–2.53)	5	1.11	(0.53–2.33)
Thyroid (C73.9)	10	3.01	(1.35–6.73)	10	1.28	(0.57–2.85)
Oral (C00-C14)	10	1.16	(0.62–2.15)	5	2.70	(1.28–5.68)
Lip (C00.0-C00.9)	5	2.91	(1.08–7.80)	<5	-	-
Stomach (C16)	10	1.22	(0.63–2.35)	<5	-	-
Bladder (C67)	10	0.72	(0.40–1.31)	<5	-	-
Kidney (C64.9)	5	0.57	(0.26–1.27)	<5	-	-
Leukemia^c^	5	0.82	(0.37–1.83)	<5	-	-
Pancreas (C25)	<5	-	-	5	1.14	(0.51–2.54)
Cervix (C53)	-	-	-	5	1.55	(0.70–3.46)
Larynx (C32)	5	1.46	(0.61–3.52)	0	-	-
Multiple Myeloma^c^	<5	-	-	<5	-	-
Esophagus (C15)	<5	-	-	<5	-	-
Liver (C22.0, C22.1)	<5	-	-	<5	-	-
Brain (C70-C72)	<5	-	-	<5	-	-
Nasal (C30)	<5	-	-	0	-	-
Bone (C40, C41)	<5	-	-	0	-	-
Hodgkin Lymphoma^c^	<5	-	-	0	-	-
Ovary (C56.9)	-	-	-	<5	-	-
Testis (C62)	0	-	-	-	-	-
Mesothelioma^c^	0	-	-	0	-	-

Note: case counts below 5 have been suppressed and all counts have been randomly rounded to base 5 in accordance with Statistics Canada disclosure rules

^a^Adjusted for age at baseline (age group categories), province of residence at baseline, and education level at baseline

^b^Incident primary cancers excluding non-melanoma skin cancer

^c^Cancers defined using ICD-O-3 Histology codes: Mesothelioma (9050–9055), Hodgkin lymphoma (9650–9667); non-Hodgkin lymphoma (9590–9596,9670–9719, 9727–9729, 9823, 9827); Multiple myeloma (9731,9732,9734); Leukemia (9733, 9742, 9800–9801, 9805, 9820, 9826, 9831–9837, 9840, 9860–9861, 9863, 9866–9867, 9870–9876,9891,9895–9897,9910, 9920, 9930–9931, 9940, 9945–9946, 9948, 9963–9964, 9823, 9827)

### Sensitivity analyses

In an effort to refine the pattern of cancer risks observed for agricultural workers, we carried out several stratified analyses. First, a sub-cohort was created by excluding individuals with a cancer diagnosis within 10 years of cohort inception, between 1981 and 1991. These exclusions removed approximately 28,680 individuals from the entire working cohort, including prevalent cases and individuals with a past history of cancer. Associations with agricultural occupations were estimated separately for men (Additional file 1: Table S1) and women (Additional file 2: Table S2).

The pattern and magnitude of cancer risks among agricultural workers in the cancer-free sub-cohort closely resembled those observed in the main analysis. Risk of oral cavity cancers was only elevated in the labourer group (HR = 1.25, 95% CI: 1.00–1.57). However, an approximately 2-fold increase in lip cancer risk was observed for all male agricultural workers (HR = 2.10, 95% CI: 1.65–2.66), farmers and managers (HR = 2.17, 95% CI: 1.67–2.82) and manual labourers (HR = 1.85, 95% CI: 1.18–2.92). The previously observed 11% and 12% increase in prostate cancer risk overall, and among farmers and managers, persisted in the cancer-free sub-cohort. A similar pattern was observed for melanoma, with 15% greater risks observed for agricultural work overall and a 21% increase in risk observed in farmers and managers. Although based on a small number of cases, the risk of male breast cancer was more than double among workers and labourers in agriculture (HR = 2.14, 95% CI: 1.00–4.58).

The exclusion of pre-1991 cancer cases did not alter the pattern of associations observed for women employed in agriculture (Additional file 2: Table S2). The increase in leukemia risk was the highest among farmers and managers (HR = 1.74, 95% CI: 1.29–2.35), and somewhat attenuated in the overall exposure group (HR = 1.24, 95% CI: 0.98–1.58). Risk of pancreatic cancer was 42% greater among female labourers and 32% greater for agricultural workers overall. Excess risks of thyroid cancer were observed for all women in agriculture (HR = 1.26, 95% CI: 0.99–1.60), and brain cancer incidence appeared elevated among female farmers and managers (HR = 1.45, 95% CI: 0.96–2.21).

In our next sensitivity analysis, we estimated risks associated with agricultural occupation for selected cancers separately among participants aged 25 to 44 years old at enrollment and those aged 45 to 74 years on census day (Additonal file 3: Table S3). As expected, the number of cancers diagnosed in the older age group (7945 cases) was higher than the number of cases observed among younger participants (1570 cases). Increased risks of leukemia (HR = 1.30, 95% CI: 1.15–1.47), multiple myeloma (HR = 1.22, 95% CI: 1.01–1.46), prostate (HR = 1.26, 95% CI: 1.21–1.32) and thyroid cancer (HR = 1.34, 95% CI: 1.07–1.72) were limited to agricultural workers in the older age stratum.

However, the increased risk of lip cancer identified in the main analyses was observed among agricultural workers aged 25 to 44 years (HR = 2.38, 95% CI: 1.32–4.28), as well as in the older age stratum (HR = 2.28, 95% CI: 1.79–2.91). Melanoma risks were only 8% greater among younger participants, but risks associated with agricultural work were significantly elevated by 25% among workers aged 45 to 74 years. Excess risk of NHL was observed among agricultural workers aged 45 to 74 years (HR = 1.19, 95% CI: 1.08–1.30) and this association was attenuated in the younger age group (HR = 1.14, 95% CI: 0.95–1.37).

## Discussion

This analysis of a large, population-based cohort study of Canadian agricultural workers has confirmed many established patterns of cancer risk, and uncovered several novel associations. As expected, overall cancer risk was significantly lower for both men and women employed in agricultural occupations, in comparison with the rest of the working population. The incidence of main tobacco- and alcohol-related cancers, such as lung, liver and larynx was also significantly reduced for both men and women working in agriculture.

However, a cautious interpretation of our findings is warranted since this cohort was designed for the purposes of cancer surveillance, with the primary goal of identifying meaningful patterns in cancer incidence by occupation. Therefore, while this analysis sacrifices some detail and specificity compared to exposure assessments in studies designed to evaluate specific agricultural exposures, linkage projects such as this one provide a unique opportunity to examine cancer patterns by occupation for a large, nationally representative sample of the population. Although our findings do not elucidate specific exposure-response relationships, the associations identified in this cohort provide leads regarding exposures that may be implicated in cancer risk and warrant follow-up in other studies.

Pesticides, several of which have been classified as known (Group 1) or probable (Group 2A) human carcinogens by the International Agency for Research on Cancer and several regulatory agencies in the United States [23–25], are among the most prevalent and studied agricultural exposures [26]. Exposure to pesticides is often considered a major factor underlying increased risks of NHL, multiple myeloma, and leukemia observed in agricultural populations [3, 11]. A meta-analysis of 13 case-control studies observed a significantly increased risk of NHL associated with occupational pesticide exposure, and suggestive associations were reported for other hematopoietic cancers [27]. This pattern of results is consistent with the findings of population-based case-controls studies of pesticide exposure in Canada [28–30] and the United States [31–33], as well as results from the AHS cohort [10].

Our findings of increased leukemia risk among female farmers and managers, and specifically crop farmers, are also supported by recent studies of farming and pesticide exposure. The Iowa Women’s Health Study, a large cohort created by linkage to the Iowa Cancer Registry, reported increased risks of acute myeloid leukemia (AML) among women living on farms [34]. Furthermore, a large multi-site prospective cohort of postmenopausal women in the United States also reported increased risks of chronic lymphocytic leukemia/small lymphocytic lymphoma and AML associated with insecticide exposure [35]. Therefore, while the excess risks of hematological cancers observed in our study cannot be directly attributed to a specific exposure, the existing epidemiologic literature is suggestive of pesticides as a contributing factor.

The increased risk of pancreatic cancer observed among women employed in agriculture is a noteworthy finding of this study. A Spanish case-control study observed similarly increased risks of pancreatic cancer among female, but not male, agricultural workers [36]. However, a later analysis of a similar population-based case-control study in Spain observed a non-significant suggestive increase in risk male agricultural workers only [37]. A meta-analysis of occupational risk factors for pancreatic cancer identified several positive but weak associations, with the strongest evidence observed for nickel compounds based on results in four populations [38].

Pesticide exposure has been linked to prostate cancer [39, 40], however, the overall evidence for an increased risk of prostate cancer in farmers remains weak [41] and is unlikely to account for the modestly increased risks observed in our study. The observed associations may reflect a multitude of factors, including genetic predisposition and screening behavior, and should be interpreted with caution since the etiology of prostate cancer is poorly understood. Currently, the only established risk factors for prostate cancer are age, ethnicity, and a positive family history of prostate cancer [42]. Despite extensive research into lifestyle factors, the epidemiological evidence remains mixed. Early observations of increased prostate cancer risk associated with reduced sunlight exposure prompted investigations into the protective effects of vitamin D [43]. Although it was initially promoted for prostate cancer prevention [44], the accumulation of convicting findings, and recent reviews and meta-analyses of vitamin D do not support a convincing causal relationship with prostate cancer incidence [45–47].

The inverse risk estimates observed for kidney, colon, rectal and bladder cancers are suggestive of a favourable risk factor profile in agricultural workers with respect to physical activity and body weight, which are recognized modifiable risk factors for these cancers [48, 49]. The decreased risks of bladder cancer observed consistently among male agricultural workers may also be related to the low prevalence of cigarette smoking in this population [50]. However, the impact of other environmental and occupational bladder cancer risk factors, such as contamination of drinking water with arsenic, exposure to polycyclic aromatic hydrocarbons (PAHs), aromatic amines, and diesel and gasoline emissions cannot be discounted [51–53].

In addition to a low prevalence of tobacco smoking, endotoxin exposure may also contribute to the lower risk of lung cancer observed in agricultural workers. Endotoxin, or lipopolysaccharide (LPS), is a component of the outer membrane of Gram-negative bacteria, and is released during cell replication. These molecules are ubiquitous and inhalation of endotoxins present in dust is the main route of exposure. High levels of endotoxin exposure have been documented in agricultural settings, especially for activities involving animal breeding and handling [54]. Although the epidemiologic studies do not provide strong causal evidence, some mechanistic studies suggest that endotoxins can inhibit tumor initiation and growth, and LPS may stimulate the production of endogenous antineoplastic mediators [55–57]. A systematic review of cohort and case control studies of lung cancer in cotton textile production and agriculture found significant inverse associations for endotoxin exposure among livestock farmers [58], suggesting that it may partly contribute to the lower risk of lung cancer observed for some agricultural occupations.

Our findings of statistically significant increases in the risk of lip cancer and melanoma among men implicate exposure to sunlight and ultraviolet (UV) radiation as a putative risk factor. Studies have identified UV exposure as a major determinant of both melanoma and non-melanoma skin cancers, including cancers of the facial skin [59]. Due to the small number of lip cancers in women this association could not be estimated, although excesses in melanoma risk, especially among female crop farmers, are compatible with this hypothesis. Overall, it appears that occupations associated high socioeconomic status, as well as jobs that involve outdoor work and potential for exposure to industrial chemicals tend to show increased risks of skin and lip cancer, compared with the general population [7, 60]. However, increased melanoma risk among agricultural workers may also be related to pesticide exposure, with studies reporting associations both with occupational [61] and residential use [62].

Cancers of the oral cavity are a diverse group of tumours arising from the epithelium lining the oral cavity and pharynx, with distinct risk factor profiles. The increased risks observed for lip cancer specifically, rather than all oral cavity cancers, is likely to reflect the interplay between risk factors shared common to all oral carcinomas, such as alcohol consumption and tobacco smoking, and more specific causal factors, such as human papillomavirus infection, a susceptibility factor for oropharyngeal carcinoma [63, 64].

Our analysis of more refined agricultural subgroups also revealed a 3-fold increased risk of thyroid cancer among male crop farmers. There are few known thyroid cancer risk factors with the exception of female sex and ionizing radiation. Although thyroid hormone excretion and metabolism can be disrupted by a number of chemicals found in the workplace, such as organochlorine pesticides, polychlorinated biphenyls, and polybrominated diphenyl ethers, the link with thyroid cancer remains tenuous. An analysis of predominantly male atrazine users in the AHS found an elevated risk of thyroid cancer for the highest compared to the lowest category of intensity-weighted use, but the trend was not monotonic and not statistically significant [65].

Several limitations of this analysis should be acknowledged. Given the retrospective nature of this cohort and its creation using linkage between existing health and administrative data sources, we did not have information on duration of employment in agriculture prior to the 1991 Census. We were also unable to track changes in occupation over the course of the follow-up period. This relatively crude approach is likely to reduce the specificity and sensitivity of our exposure assessment, resulting in exposure misclassification. However, since these limitations apply to all occupational groups within the cohort, and errors in exposure ascertainment are independent of cancer status, comparisons between agricultural workers and other groups may lead to imprecise risk estimates, but are unlikely to produce spurious associations [66]. Furthermore, classifying individuals with a short duration of employment in agriculture as exposed, would be expected to bias associations towards the null.

A second limitation concerns the possibility that some participants were diagnosed with cancer prior to cohort inception in 1991. Failure to account for a past history of cancer would only bias our analyses if agricultural workers as a group were enriched for cancer survivors, compared to all other occupations. However, since this is unlikely, a more relevant concern is that some prevalent cancer cases in the cohort were classified as outcome-free. This would be expected to bias the observed associations towards the null if the misclassification of disease status is non-differential with respect to occupation. While we acknowledge that a pre-1991 cancer diagnosis may affect the participant’s subsequent employment trajectory and occupation reported on census day, these effects are unlikely to have a differential impact on workers in agriculture compared to other industries [67].

Furthermore, our sensitivity analyses demonstrate that most of the observed associations persisted after the exclusion of individuals diagnosed with cancer within 10 years of cohort inception. The excess risks of lip cancer, melanoma and NHL in men were also observed in the sub-cohort, along with the inverse associations for lung, liver, bladder and larynx cancers. Similarly, the array of increased and decreased risks observed for female agricultural workers in the main analysis were also observed in the sensitivity analyses. Therefore, the stable pattern of associations emerging in both the full CanCHEC cohort and the cancer-free sub-cohort suggests that the inclusion of pre-1991 cancer cases is unlikely to bias the observed cancer risks or create spurious associations.

Confounding by lifestyle-related cancer risk factors is often a concern in occupational studies, as well as in studies relying on linkages between cancer registries and existing databases. Although smoking information was not available in CanCHEC, previous studies, in addition to analyses using the same linked 1991 Census data show that major determinants of smoking status include age, sex, level of education and occupation [21, 22]. Therefore, although confounding by smoking cannot be excluded, adjustment for key determinants of smoking status in our analysis helps mitigate the impact of this residual confounding. Furthermore, the magnitude of confounding by tobacco smoking or high levels of alcohol consumption may be further minimized in our study due to the low prevalence of both of these risk factors in agricultural populations, contemporaneous to CanCHEC inception [68, 69].

Other limitations of this study include limited power for investigating less common cancers, especially within more refined agricultural subgroups. In addition, given the large number of cancer sites and occupational subgroups that were tested, some of the observed associations may be chance findings due to multiple comparisons.

Our study has a number of important strengths. CanCHEC is the largest population-based cohort in Canada, and one of the largest studies of its kind worldwide. Although it is smaller than the previously mentioned NOCCA cohort of 15 million [7], the 2.1 million participants in CanCHEC, including 70,570 agricultural workers are broadly representative of the Canadian population due to the high response rate for the 1991 Census [15]. Although the exposure assessment is less detailed than in studies such as the AHS, CanCHEC provides a powerful resource for examining cancer patterns in specific population groups and represents a valuable addition to the cancer literature by demonstrating that disparities in cancer risk by occupation status continue to persist and should be investigated using more targeted studies. The updated linkage to the Canadian Cancer Registry allows for a long follow-up period, which is important for cancer outcomes that have long latency periods. Together, the large sample size and length of follow-up allow for a large number of events, which results in improved statistical power for the investigation of detailed occupational subgroups and specific cancer sites. Importantly, relatively few studies focus on women employed in agriculture, and even fewer have systematically reported estimates of cancer risk across multiple sites and occupational subgroups.

In the beginning twentieth century, agriculture was the single most common occupation, employing over 1 million Canadians and accounting for one-third of all jobs. Over the course of our follow-up period, between 1991 and 2011, the total number of Canadian farms fell by over 74,000 and now represents less than 1% of the labour force. However, if agricultural populations experience higher incidence for certain types of cancer, understanding the magnitude of these increased risks represents an important step towards developing preventive efforts targeting these populations.

Despite the changing profile of Canadian agricultural workers, studies of this population continue to be relevant by providing insight into cancer risks associated with common exposures that are often found outside of agricultural settings. For instance, the large magnitude of UV-related risks, especially for lip cancer, underscores the substantial impact that may be achieved by preventive efforts in this area. Therefore, studies of agricultural workers can inform preventive interventions aimed at reducing the cancer burden in the general population.

## Conclusions

In summary, the results of the present analysis point to excess risks for certain cancers among agricultural workers. A wide range of exposures is possible in agriculture, including pesticides, solvents, engine exhaust emission, UV light, dust, as well as zoonotic viruses and bacteria. Exposures can vary considerably between occupations, and even between farms, therefore future research must focus on specific exposures to identify and clarify which risk factors may contribute to the observed pattern of cancer incidence.

## Additional files

Additional file 1: Table S1. Hazard ratios (HR) and 95% confidence intervals (CI) for selected cancers among male agricultural workers in a sub-cohort of CanCHEC that excludes all individuals with a cancer diagnosis within 10 years of cohort inception (1981–1991). (PDF 84 kb)

Additional file 2: Table S2. Hazard ratios (HR) and 95% confidence intervals (CI) for selected cancers among female agricultural workers in a sub-cohort of CanCHEC that excludes all individuals with a cancer diagnosis within 10 years of cohort inception (1981–1991). (PDF 84 kb)

Additional file 3: Table S3. Hazard ratios (HR) and 95% confidence intervals (CI) for selected cancers with more than 100 cases among agricultural workers in CanCHEC (1991-2010), stratified by age at enrollment in 1991 (1981–1991). (PDF 53 kb)

## Abbreviations

AHS: Agricultural health study
AML: Acute myeloid leukemia
CanCHEC: Canadian Census Health and Environment Cohort
CCR: Canadian cancer registry
CMDB: Canadian mortality database
HR: Hazard ratio
CI: Confidence intervals
IARC: International Agency for Research on Cancer
ICD-O: International classification of diseases for oncology
LPS: Lipopolysaccharide
NHL: Non-Hodgkin lymphoma
SOC: Standard occupational classification
UV: Ultraviolet radiation
