# Supplementary material for: Cancer risks in a population-based study of 70,570 agricultural workers: results from the Canadian census health and Environment cohort (CanCHEC)
Source: BMC Cancer. 2017 May 19;17:343. doi: 10.1186/s12885-017-3346-x (PMC5437486; doi:10.1186/s12885-017-3346-x)
Supplement: Supplementary file 1 — Hazard ratios (HR) and 95% confidence intervals (CI) for selected cancers among male agricultural workers in a sub-cohort of CanCHEC that excludes all individuals with a cancer diagnosis within 10 years of cohort inception (1981–1991). (PDF 84 kb) [file 12885_2017_3346_MOESM1_ESM.pdf]

Table S1: Hazard ratios (HR) and 95% confidence intervals (CI) for selected cancers among male agricultural workers in a sub-cohort of CanCHEC that excludes all individuals with a cancer diagnosis within 10 years of cohort inception (1981-1991)

Cancer Site (ICD-O-3)		Agricultural Workers		Farmers and managers		Manual labourers	
		HR	(95% CI)[1]	HR	(95% CI)[1]	HR	(95% CI)[1]
Any cancer[2]		0.95	(0.93-0.98)	0.94	(0.92-0.97)	0.98	(0.94-1.03)
Prostate	(C61.9)	1.11	(1.06-1.16)	1.12	(1.07-1.18)	1.07	(0.99-1.17)
Lung	(C34)	0.75	(0.70-0.80)	0.70	(0.64-0.75)	0.91	(0.81-1.02)
Colon	(C18, C26.0)	0.89	(0.82-0.97)	0.89	(0.81-0.99)	0.89	(0.75-1.04)
Rectum	(C19.9, C20.9)	1.05	(0.94-1.16)	1.09	(0.97-1.22)	0.92	(0.75-1.13)
Non-Hodgkin Lymphoma[3]		1.11	(1.00-1.22)	1.11	(0.99-1.24)	1.10	(0.92-1.32)
Bladder	(C67)	0.82	(0.74-0.91)	0.82	(0.73-0.93)	0.80	(0.66-0.98)
Melanoma	(C44)	1.15	(1.01-1.31)	1.21	(1.04-1.40)	1.00	(0.77-1.29)
Leukemia[3]		1.10	(0.96-1.26)	1.12	(0.96-1.31)	1.05	(0.82-1.35)
Oral	(C00-C14)	1.04	(0.91-1.19)	0.96	(0.81-1.13)	1.25	(1.00-1.57)
Lip	(C00.0-C00.9)	2.10	(1.65-2.66)	2.17	(1.67-2.82)	1.85	(1.18-2.92)
Kidney	(C64.9)	0.77	(0.67-0.89)	0.79	(0.67-0.92)	0.72	(0.55-0.95)
Stomach	(C16)	0.91	(0.79-1.05)	0.87	(0.74-1.04)	1.01	(0.79-1.29)
Pancreas	(C25)	0.92	(0.78-1.09)	0.88	(0.72-1.06)	1.05	(0.79-1.40)
Multiple myeloma[3]		1.12	(0.93-1.36)	1.11	(0.89-1.39)	1.15	(0.82-1.61)
Brain	(C70-C72)	0.87	(0.71-1.07)	0.81	(0.64-1.04)	1.02	(0.73-1.42)
Esophagus	(C15)	0.86	(0.69-1.06)	0.77	(0.59-0.99)	1.12	(0.78-1.62)
Thyroid	(C73.9)	0.95	(0.71-1.28)	0.95	(0.68-1.34)	0.95	(0.55-1.64)
Larynx	(C32)	0.53	(0.40-0.70)	0.39	(0.27-0.57)	0.90	(0.59-1.36)
Liver	(C22.0, C22.1)	0.51	(0.38-0.68)	0.42	(0.29-0.61)	0.76	(0.48-1.21)
Testis	(C62)	0.96	(0.68-1.36)	1.00	(0.64-1.55)	0.91	(0.53-1.58)
Hodgkin Lymphoma[3]		0.76	(0.47-1.23)	0.68	(0.37-1.25)	0.92	(0.43-1.94)
Mesothelioma[3]		0.58	(0.37-0.92)	0.57	(0.34-0.97)	0.61	(0.25-1.47)
Breast	(C50)	1.32	(0.78-2.25)	1.01	(0.50-2.01)	2.14	(1.00-4.58)
Nasal	(C30)	0.72	(0.40-1.32)	0.79	(0.39-1.58)	0.60	(0.22-1.67)
Bone	(C40, C41)	0.70	(0.36-1.35)	-	-	-	-

[1] Adjusted for age at baseline (age group categories), province of residence at baseline, and education level at baseline
[2] Incident primary cancers excluding non-melanoma skin cancer
[3] Cancers defined using ICD-O-3 Histology codes: Mesothelioma (9050–9055), Hodgkin lymphoma (9650–9667); non-Hodgkin lymphoma (9590–9596,9670–9719, 9727–9729, 9823, 9827); Multiple myeloma (9731,9732,9734); Leukemia (9733, 9742, 9800–9801, 9805, 9820, 9826, 9831–9837, 9840, 9860–9861, 9863, 9866–9867, 9870–9876,9891,9895–9897,9910, 9920, 9930–9931, 9940, 9945–9946, 9948, 9963–9964, 9823, 9827)

Note: case counts below 5 have been suppressed and all counts have been randomly rounded to base 5 in accordance with Statistics Canada disclosure rules
