# Supplementary material for: Cancer risks in a population-based study of 70,570 agricultural workers: results from the Canadian census health and Environment cohort (CanCHEC)
Source: BMC Cancer. 2017 May 19;17:343. doi: 10.1186/s12885-017-3346-x (PMC5437486; doi:10.1186/s12885-017-3346-x)
Supplement: Supplementary file 2 — Hazard ratios (HR) and 95% confidence intervals (CI) for selected cancers among female agricultural workers in a sub-cohort of CanCHEC that excludes all individuals with a cancer diagnosis within 10 years of cohort inception (1981–1991). (PDF 84 kb) [file 12885_2017_3346_MOESM2_ESM.pdf]

Table S2: Hazard ratios (HR) and 95% confidence intervals (CI) for selected cancers among female agricultural workers in a sub-cohort of CanCHEC that excludes all individuals with a cancer diagnosis within 10 years of cohort inception (1981-1991)

Cancer Site (ICD-O-3)		Agricultural Workers		Farmers and managers		Manual labourers	
		**HR**	**(95% CI)**[1]	**HR**	**(95% CI)**[1]	**HR**	**(95% CI)**[1]
Any cancer[2]		0.92	(0.88-0.96)	0.96	(0.90-1.02)	0.88	(0.83-0.84)
Breast	(C50)	0.93	(0.86-1.00)	0.93	(0.83-1.03)	0.92	(0.83-1.03)
Lung	(C34)	0.58	(0.50-0.66)	0.56	(0.46-0.69)	0.59	(0.49-0.72)
Colon	(C18, C26.0)	0.94	(0.82-1.09)	1.20	(1.00-1.44)	0.71	(0.56-0.89)
Non-Hodgkin Lymphoma[3]		1.04	(0.87-1.23)	1.18	(0.93-1.49)	0.91	(0.71-1.17)
Rectum	(C19.9, C20.9)	1.04	(0.84-1.28)	1.03	(0.76-1.39)	1.05	(0.79-1.39)
Melanoma	(C44)	1.15	(0.93-1.42)	1.02	(0.74-1.42)	1.26	(0.96-1.65)
Ovary	(C56.9)	1.01	(0.81-1.26)	0.92	(0.66-1.28)	1.09	(0.81-1.47)
Leukemia[3]		1.24	(0.98-1.58)	1.74	(1.29-2.35)	0.84	(0.58-1.23)
Thyroid	(C73.9)	1.26	(0.99-1.60)	1.23	(0.86-1.76)	1.28	(0.94-1.75)
Pancreas	(C25)	1.32	(1.03-1.69)	1.23	(0.88-1.74)	1.42	(1.01-1.98)
Bladder	(C67)	0.90	(0.67-1.20)	0.86	(0.56-1.33)	0.92	(0.92-1.37)
Kidney	(C64.9)	0.79	(0.58-1.08)	0.86	(0.57-1.30)	0.71	(0.45-1.14)
Brain	(C70-C72)	1.26	(0.92-1.72)	1.45	(0.96-2.21)	1.08	(0.69-1.71)
Cervix	(C53)	0.82	(0.59-1.14)	0.71	(0.42-1.20)	0.91	(0.60-1.38)
Stomach	(C16)	0.92	(0.64-1.31)	1.18	(0.75-1.85)	0.67	(0.38-1.92)
Oral	(C00-C14)	0.77	(0.53-1.11)	0.73	(0.44-1.22)	0.81	(0.48-1.38)
Lip	(C00.0-C00.9)	-	-	-	-	-	-
Multiple myeloma[3]		0.81	(0.54-1.21)	0.51	(0.27-0.95)	1.30	(0.78-2.18)
Liver	(C22.0, C22.1)	0.93	(0.53-1.63)	0.91	(0.43-1.95)	0.95	(0.42-2.15)
Esophagus	(C15)	1.10	(0.62-1.94)	1.47	(0.69-3.14)	0.84	(0.37-1.92)
Mesothelioma[3]		-	-	-	-	-	-
Larynx	(C32)	-	-	-	-	-	-
Nasal	(C30)	-	-	-	-	-	-
Bone	(C40, C41)	-	-	-	-	-	-
Hodgkin Lymphoma[3]		-	-	-	-	-	-

[1] Adjusted for age at baseline (age group categories), province of residence at baseline, and education level at baseline
[2] Incident primary cancers excluding non-melanoma skin cancer
[3] Cancers defined using ICD-O-3 Histology codes: Mesothelioma (9050–9055), Hodgkin lymphoma (9650–9667); non-Hodgkin lymphoma (9590–9596,9670–9719, 9727–9729, 9823, 9827); Multiple myeloma (9731,9732,9734); Leukemia (9733, 9742, 9800–9801, 9805, 9820, 9826, 9831–9837, 9840, 9860–9861, 9863, 9866–9867, 9870–9876,9891,9895–9897,9910, 9920, 9930–9931, 9940, 9945–9946, 9948, 9963–9964, 9823, 9827)

Note: case counts below 5 have been suppressed and all counts have been randomly rounded to base 5 in accordance with Statistics Canada disclosure rules
