# Supplementary material for: Cancer risks in a population-based study of 70,570 agricultural workers: results from the Canadian census health and Environment cohort (CanCHEC)
Source: BMC Cancer. 2017 May 19;17:343. doi: 10.1186/s12885-017-3346-x (PMC5437486; doi:10.1186/s12885-017-3346-x)
Supplement: Supplementary file 3 — Hazard ratios (HR) and 95% confidence intervals (CI) for selected cancers with more than 100 cases among agricultural workers in CanCHEC (1991-2010), stratified by age at enrollment in 1991 (1981–1991). (PDF 53 kb) [file 12885_2017_3346_MOESM3_ESM.pdf]

Supplementary Table S3: Hazard ratios (HR) and 95% confidence intervals (CI) for selected cancers with more than 100 cases among agricultural workers in CanCHEC (1991-2010), stratified by age at enrollment in 1991

**Cancer Site (ICD-O-3)**		**25 – 44 years**			**45 – 74 years**		
		**Cases**	**HR**	**(95% CI)[1]**	**Cases**	**HR**	**(95% CI)[1]**
Any cancer[2]		1570	0.91	(0.87-0.96)	7945	1.05	(1.03-1.08)
Prostate	(C61.9)	200	0.89	(0.77-1.03)	2430	1.26	(1.21-1.32)
Lung	(C34)	140	0.78	(0.66-0.92)	1065	0.77	(0.72-0.82)
Colon	(C18, C26.0)	105	0.86	(0.70-1.05)	735	1.03	(0.95-1.11)
Breast	(C50)	230	0.92	(0.80-1.04)	490	0.94	(0.86-1.03)
Non-Hodgkin Lymphoma[3]		120	1.14	(0.95-1.37)	510	1.19	(1.08-1.30)
Rectum	(C19.9, C20.9)	85	1.08	(0.87-1.35)	450	1.15	(1.04-1.27)
Bladder	(C67)	45	0.67	(0.49-0.90)	435	0.97	(0.88-1.08)
Melanoma	(C44)	115	1.08	(0.90-1.31)	250	1.25	(1.09-1.42)
Leukemia[3]		50	0.98	(0.74-1.32)	290	1.30	(1.15-1.47)
Oral	(C00-C14)	60	0.88	(0.69-1.14)	220	1.10	(0.95-1.27)
Lip	(C00.0-C00.9)	10	2.38	(1.32-4.28)	100	2.28	(1.79-2.91)
Kidney	(C64.9)	55	0.62	(0.47-0.82)	215	0.87	(0.75-1.00)
Stomach	(C16)	30	0.76	(0.52-1.12)	230	1.06	(0.92-1.21)
Pancreas	(C25)	30	0.78	(0.54-1.13)	215	1.13	(0.97-1.30)
Multiple Myeloma[3]		25	1.04	(0.69-1.59)	135	1.22	(1.01-1.46)
Brain	(C70-C72)	50	1.12	(0.84-1.50)	105	0.99	(0.81-1.22)
Thyroid	(C73.9)	50	0.99	(0.75-1.30)	75	1.34	(1.07-1.72)

[1] Adjusted for sex, province of residence at baseline, and education level at baseline
[2] Incident primary cancers excluding non-melanoma skin cancer
[3] Cancers defined using ICD-O-3 Histology codes: Mesothelioma (9050–9055), Hodgkin lymphoma (9650–9667); non-Hodgkin lymphoma (9590–9596,9670–9719, 9727–9729, 9823, 9827); Multiple myeloma (9731,9732,9734); Leukemia (9733, 9742, 9800–9801, 9805, 9820, 9826, 9831–9837, 9840, 9860–9861, 9863, 9866–9867, 9870–9876,9891,9895–9897,9910, 9920, 9930–9931, 9940, 9945–9946, 9948, 9963–9964, 9823, 9827)

Note: case counts below 5 have been suppressed and all counts have been randomly rounded to base 5 in accordance with Statistics Canada disclosure rules
